# Low-Grade Endometrial Stromal Sarcoma with a Nodule-in-Nodule Appearance in Preoperative Magnetic Resonance Images

**DOI:** 10.1155/2020/8973262

**Published:** 2020-07-30

**Authors:** Mitsuhiro Nakamura, Ryusuke Murakami, Kaoru Abiko, Taito Miyamoto, Yoshimi Kitawaki, Ken Yamaguchi, Akihito Horie, Junzo Hamanishi, Eiji Kodoh, Tsukasa Baba, Aki Kido, Sachiko Minamiguchi, Noriomi Matsumura, Masaki Mandai

**Affiliations:** ^1^Department of Gynecology and Obstetrics, Kyoto University Graduate School of Medicine, Kyoto, Japan; ^2^Department of Obstetrics and Gynecology, Japanese Red Cross Society Wakayama Medical Center, Wakayama, Japan; ^3^Department of Gynecology, Shiga General Hospital, Shiga, Japan; ^4^Department of Obstetrics and Gynecology, National Hospital Organization Kyoto Medical Center, Kyoto, Japan; ^5^Department of Obstetrics and Gynecology, Iwate Medical University Faculty of Medicine, Iwate, Japan; ^6^Department of Diagnostic Imaging and Nuclear Medicine, Kyoto University Graduate school of Medicine, Kyoto, Japan; ^7^Department of Diagnostic Pathology, Kyoto University Graduate School of Medicine, Kyoto, Japan; ^8^Department of Obstetrics and Gynecology, Kindai University Hospital, Osaka, Japan

## Abstract

Low-grade endometrial stromal sarcoma (LG-ESS) is a rare malignant disease and demonstrates various patterns in preoperative imaging. Therefore, accurate diagnosis is important. Given its unique form, we report a case of LG-ESS with a nodule-in-nodule appearance on preoperative imaging. A 41-year-old woman was referred to our department for further examination of a 45 mm diameter uterine corpus mass. Preoperative magnetic resonance imaging (MRI) revealed several small nodules within a larger nodule. T2-weighted images showed moderate-to-high signal intensity with focal bands of low signal intensity in the small nodules. The patient underwent total abdominal hysterectomy and bilateral salpingo-oophorectomy. Histopathological findings of the small nodules showed densely concentrated endometrial stromal cells reminiscent of a proliferative phase endometrium with a concentric arrangement of small spiral arteriole-like vessels. The small nodules exhibited an expansile growth pattern and were surrounded by less densely concentrated endometrial stromal cells intermingled with the normal uterine myometrium. LG-ESS with smooth muscle differentiation and sex cord-like elements was partially observed. In summary, LG-ESS demonstrating a unique nodule-in-nodule appearance on preoperative imaging histopathologically comprised tumor cells of varying densities. Our current case suggests that preoperative diagnostic imaging with MRI may be useful.

## 1. Introduction

Endometrial stromal sarcoma (ESS) has a reported frequency of approximately 3%–9% of uterine malignancies and 14% of uterine sarcomas [1]. Low-grade ESS (LG-ESS) presents with diverse forms of differentiation. The characteristic presentation of LG-ESS in magnetic resonance imaging (MRI) findings is a tumor that appears as a polypoid endometrial mass with hypointensity at T1-weighted images (WI) and heterogeneous hyperintensity at T2-WI. It typically shows both endometrial involvement and myometrial involvement, either sharply demarcated or more diffuse with a tendency of lymphatic and vascular invasions. Bands of low signal intensity are observed within the area of tumor at T2-WI, which is reported to be a pathologically preserved myometrial bundle left behind by the worm-like extension of the tumor [2–6]. We report our experience of a patient whose MRI scans exhibited unique characteristics of a tumor with a nodule-in-nodule appearance, which was diagnosed as LG-ESS. To our knowledge, we are the first to report the preoperative MRI findings of a nodule-in-nodule pattern for LG-ESS worldwide.

## 2. Case Presentation

The patient was a 41-year-old woman (gravida 2, para 1) with no chief complaints. She experienced menarche at the age of 12 years and had a regular menstrual cycle, low menstrual volume, and no apparent dysmenorrhea. Her medical and family histories were unremarkable. During consultation with a previous physician regarding her desire to bear a child, transvaginal ultrasound revealed subserosal and submucosal myoma with degeneration. Pelvic MRI showed a component with limited diffusion in the interior of a mass measuring 45 mm in the uterine muscle layer. Uterine sarcoma was suspected, and the patient was referred to our department for further examination.

**Figure 1:**
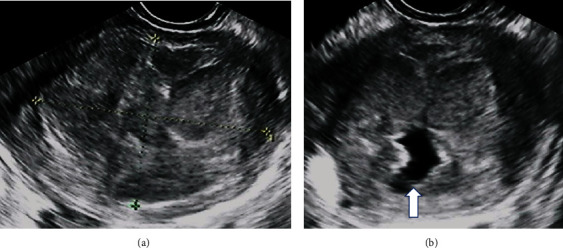
Transvaginal ultrasound images: (a) the tumor margin and the underlying myometrium were hyperechoic and well defined. Several small nodules within a large mass were isoechoic and hypoechoic compared to the normal uterine myometrium; (b) an anechoic unilocular cystic area within a small nodule was observed (indicated by the arrow).

Pelvic examination on the first visit revealed that the uterine body had a size of a man's fist and was anteflexed. Transvaginal ultrasound showed a mass in the uterine body measuring approximately 5 cm and composed of multiple nodules. The tumor margin and the underlying myometrium were hyperechoic and well defined. Several small nodules within a large mass were isoechoic and hypoechoic compared to the normal myometrium (Figure 1(a)). An anechoic unilocular cystic area within a small nodule was observed (Figure 1(b)). Differential diagnosis included leiomyoma with hydropic degeneration and sarcoma, among other possibilities. Blood tests did not show elevated tumor markers, including cancer antigen 125 (CA125) = 9.7 U/mL and CA19‐9 = 3.0 U/mL.

MRI was performed using a 3 T unit. Before examination, 20 mg of butyl scopolamine (Buscopan®; Nippon Boehringer Ingelheim Co. Ltd., Tokyo, Japan) was administered to reduce bowel motion. The examination involved sagittal T1-WI (TR/TE = 608/11 ms, slice thickness 4 mm), axial and sagittal T2WI (TR/TE = 4000/81 ms, slice thickness 4 mm), and sagittal diffusion-weighted imaging (DWI) (TR/TE = 5000/49 ms, *b* value = 0.500.800, 1000 s/mm^2^, slice thickness 4 mm). Apparent diffusion coefficient (ADC) maps were automatically generated using a monoexponential decay model including all four *b* values. Slice thicknesses were 4 mm. Dynamic contrast-enhanced images were obtained as follows: TR/TE of 3.42/1.24 ms and slice thickness of 4 mm. These sequenced images were acquired at different phases (20, 40, 60, 80, 100, 120, and 180 s) relative to the administration of the contrast agent (0.2 mL/kg of gadoterate dimeglumine at a 2 mL/s rate).

**Figure 2:**
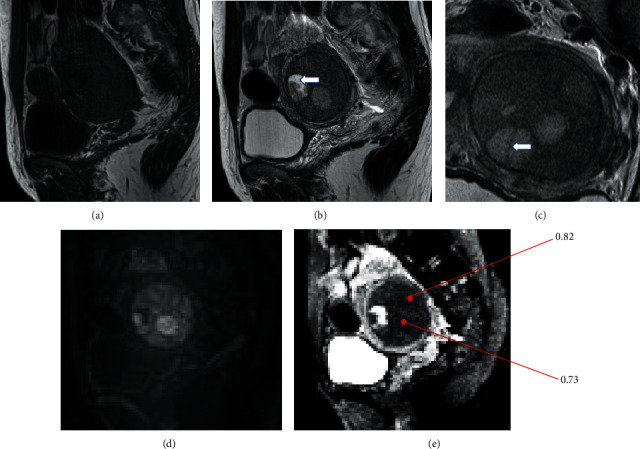
Unenhanced pelvic MRI. (a) Sagittal T1-WI shows a tumor demonstrating isointensity with the normal myometrium. (b) Sagittal T2-WI showing a tumor with a nodule-in-nodule appearance within the myometrium. The small nodules show a low signal intensity at T1-WI and a high signal intensity at T2-WI. (c) Hypointense bands within the small nodules are signalized with an arrow on coronal T2-WI. (d) At diffusion-weighted imaging (*b* = 1000 ms/mm^2^), the small nodules show a higher signal intensity than the surrounding tumors, and the surrounding tumors show a relatively higher signal intensity than the normal myometrium. (e) Both small nodules and surrounding tumors show a low signal intensity in the apparent diffusion coefficient (ADC) map. The ADC values of the small nodule and main tumor are 0.73 and 0.82 (10^−3^ mm^2^/s), respectively.

**Figure 3:**
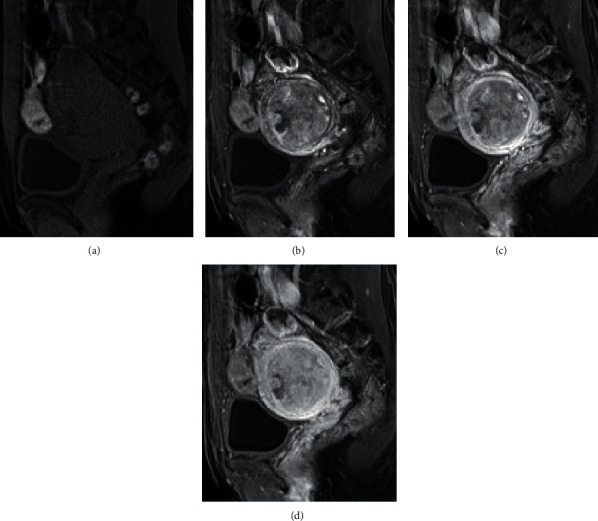
Dynamic contrast-enhanced T1-WI pelvic MRI: (a) precontrast; (b) postcontrast 20 s; (c) postcontrast 60 s; (d) postcontrast 180 s. In dynamic contrast-enhanced T1-WI, the small nodules are enhanced rapidly and stronger than the normal myometrium in the early phase and decrease gradually in the later phase to the level similar to that of the normal myometrium. In contrast, the larger mass is enhanced gradually and heterogeneously, but it is weaker than the normal myometrium.

Pelvic MRI revealed a well-defined mass with a diameter of 45 mm within the myometrium, which showed isointensity with the normal myometrium at T1-WI and lower signal intensity than the normal myometrium at T2-WI (Figures 2(a) and 2(b)). Multiple elliptical nodules with a diameter of 2 cm were also observed inside the mass showing a higher signal intensity at T2-WI than the surrounding tumor, which appeared to present a “nodule-in-nodule” appearance. Thin hypointense rims delineatheddelineathed the small nodules and the tumor at T2-WI. A high signal intense area was observed within the ventral small nodule and thought to be a cystic area (Figure 2(b)). Hypointense bands were observed within the small nodule at T2-WI (Figure 2(c)). The main tumor and most of the small nodules showed restricted water diffusion compared to the surrounding tissues, which means a high signal intensity at DWI (*b* = 1000 ms/mm^2^) (Figure 2(d)) and a low signal intensity at the apparent diffusion coefficient (ADC) map (Figure 2(e)). The ADC values of the small nodule and main tumor were 0.73 and 0.82 (10^−3^ mm^2^/s), respectively (Figure 2(e)). A part of the small nodule in the anterior part of the tumor did not show restricted diffusion (Figures 2(d) and 2(e)). In dynamic contrast-enhanced T1-WI, the larger mass enhanced gradually and heterogeneously, but it was weaker than the normal myometrium. In contrast, the small nodules were enhanced rapidly and stronger than the normal myometrium in the early phase, but they decreased gradually in the later phase to the level similar to that of the normal myometrium (Figure 3).

Suspecting LG-ESS, we performed total abdominal hysterectomy and bilateral adnexectomy. The resected uterus, with the size of a man's fist, had an egg-sized somewhat soft mass growing within the myometrium, as observed in the left uterine body.

**Figure 4:**
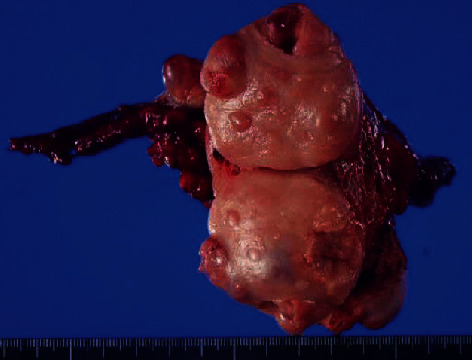
Resected specimen. Many nodules are observed on the surface of the uterine body tumor. The nodules that are harder than the soft tumor have a nodule-in-nodule appearance.

The uterus, together with both adnexa, weighed 240 g. Peritoneal lavage cytology showed no findings suggestive of malignancy. The surgically resected specimen had a large number of elastic and hard nodules within a flexible mass exhibiting a nodule-in-nodule appearance within the uterine myometrium, and the maximum diameter of the tumor was 45 mm (Figure 4).

**Figure 5:**
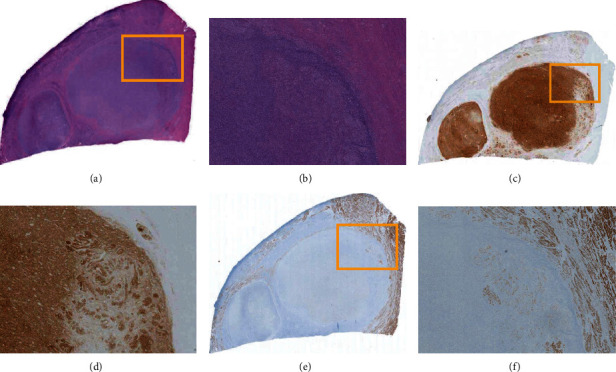
Histopathological findings of a nodule-in-nodule appearance. Microscopic imaging at loupe magnification on the left side and microscopic (×20) images on the right side corresponds to a nodule-in-nodule appearance with hematoxylin and eosin (HE) staining at the top, cluster of differentiation (CD) 10 immunohistochemistry (IHC) staining at the middle, and desmin IHC staining at the bottom. Images on the right are the magnifications of the boxed area in the left-hand images. (a) HE staining at loupe magnification. (b) HE staining at ×20 microscopic magnification. (c) CD10 IHC staining at loupe magnification. (d) CD10 IHC staining at ×20 microscopic magnification. (e) Desmin IHC staining at loupe magnification. (f) Desmin IHC staining at ×20 microscopic magnification.

Histopathologically, the mass had a nodule-in-nodule appearance when viewed microscopically, as shown in Figure 5. The figure also shows the results of hematoxylin and eosin (HE) staining, cluster of differentiation (CD) 10 immunohistochemistry (IHC) staining, and desmin IHC staining. The small nodules demonstrated a high density of CD10-positive ESS cells. Conversely, peripheral spaces between the small nodules display a mixture of CD10-negative desmin-positive smooth muscle cells with CD10-positive desmin-negative ESS cells.

**Figure 6:**
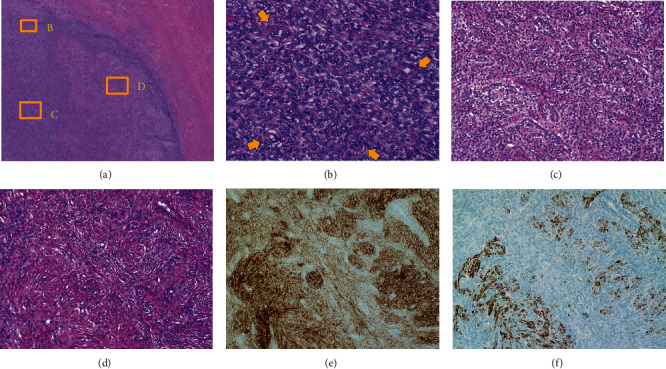
Histopathological findings of the nodule-in-nodule appearance. (a) In the small nodules, the boxed areas in Figure 5(b) (×20) are marked for further magnifications at the top left-side image. Inset squares B, C, and D are magnified in Figures 6(b) (×40), 6(c) (×20), and 6(d) (×20), respectively. (b) More densely packed endometrial stromal cells with a concentric arrangement of the small spiral arteriole-like vessels (indicated by arrows) exhibit an expansile growth pattern. (c) Sex cord-like elements are observed partially within some small nodules. (d) Peripheral areas around the small nodule are more loosely packed with endometrial stromal sarcoma (ESS) cells. Some areas are intermingled with the normal uterine myometrium. (e) ESS cells with the eosinophilic cytoplasm in hematoxylin and eosin (HE) staining are observed to be partially stained positive with cluster of differentiation (CD) 10 immunohistochemistry (IHC) markers. (f) ESS cells with the eosinophilic cytoplasm in HE staining are observed to be partially stained positive with desmin IHC markers. (d), (e), and (f) images result in the diagnosis of low-grade endometrial stromal sarcoma (LG-ESS) with smooth muscle differentiation.

At a higher magnification, in the small nodules (marked by the boxed area in Figure 5 and further magnified in Figure 6(a)), more densely packed endometrial stromal cells reminiscent of a proliferative phase endometrium with a concentric arrangement of spiral arteriole-like vessels exhibited an expansile growth pattern (Figure 6(b)), demonstrating a typical morphology of LG-ESS.

Furthermore, sex cord-like elements were partially observed (Figure 6(c)) within some small nodules. The peripheral area around the small nodules and the large nodule that showed loosely packed ESS cells comprised a part of the main tumor (Figure 6(d)). This area was intermingled with smooth muscle and ESS cells with the eosinophilic cytoplasm in HE staining. It also partially stained positive with both IHC markers CD10 (Figure 6(e)) and desmin (Figure 6(f)), which resulted in a diagnosis of LG-ESS with smooth muscle differentiation.

The diagnosis was thus specified as “LG-ESS with smooth muscle differentiation,” pT1ANxMx, and stage FIGO1A. Two years after the surgery, with no postoperative estrogen blockage administered, the patient appeared to be free of recurrent lesions.

## 3. Discussion

The current World Health Organization recognizes the following four categories of endometrial stromal tumor: endometrial stromal nodule (ESN), low-grade endometrial stromal sarcoma (LG-ESS), high-grade endometrial stromal sarcoma (HG-ESS), and undifferentiated uterine sarcoma (UUS) [7]. LG-ESS has a 5-year survival rate of 90.5%, whereas high-grade ESS (HG-ESS) has a 5-year survival rate of 32.6%, with a median survival period of 19.9 months [8]. LG-ESS is often reported as being diagnosed at an average age of 48 years, whereas HG-ESS is diagnosed at an average age of 59 years [8]. These diseases have different prognoses and clinical backgrounds, and it is important to accurately differentiate them [7, 9, 10].

Histologically, LG-ESS tumor cells resemble proliferative-phase endometrial stromal cells, and the disease is diagnosed based on the presence of invasion into the myometrium and lymph vessels. Another characteristic of LG-ESS is a relatively regular arrangement of spiral artery-like arterioles within the tumor. Given that it is difficult to assess the boundary between the tumor and normal uterine muscle in biopsy specimens, preoperative diagnosis requires careful attention. Macroscopically, LG-ESS is a tumor that shows solid growth, uterine fibroid nodules that tend to be softer and more yellowish than whitish, and hemorrhagic characteristics [7, 9, 10].

Pelvic MRI is often useful for preoperative diagnosis; however, imaging findings often resemble those of uterine fibroids, and differential diagnosis is especially difficult in cases of degenerated fibroids localized to the uterine body, and the morcellator must be handled with care. Koyama et al. reported bands of low signal intensity within the area of myometrial invasion at T2-WI as a characteristic finding of LG-ESS; these findings correspond to preserved bundles of normal myometrial fibers separated by cords or nodules of tumor cells [2]. Ueda et al. reported that half of the cases of low-grade ESS are well circumscribed, and the others show diffuse myometrial permeation by worm-like masses or multiple nodules [3]. Furukawa et al. reported that characteristic low-intensity rims at T2-WI were observed [4]. In this case, the small nodules contained areas with both restricted and nonrestricted water diffusions. The heterogeneous component was mixed within the small nodules.

In the present case, small nodules showed a typical appearance of LG-ESS, but it was difficult to interpret the surrounding tissues because they showed restricted diffusion, suggesting a malignant potential, but they did not have the same appearance with small nodules. The nodules inside a nodule on MRI were elastic firm tumors, whereas the surrounding portion appeared soft. The MRI and pathological findings were consistent with the nodule-in-nodule appearance, which was attributed to the difference in the density of LG-ESS cells and the mixed components. The small and large nodules comprised the main tumor; however, the difference was that the peripheral areas surrounding the small nodules were less densely packed with ESS cells, while the small nodules themselves were more densely packed. The areas surrounding the small nodules showed loosely packed ESS cells intermingled with normal uterine myometrium and partial smooth muscle differentiation. The magnified area in the box in Figure 5 shows the loosely packed area, which corresponds to the nonrestricted diffusion area in the small nodule in Figures 2(d) and 2(e). This was the first report to give a detailed explanation of the LG-ESS nodule-in-nodule appearance from both MRI and pathological viewpoints.

Our patient who underwent complete surgical resection in the IA stage of LG-ESS received no additional treatment and experienced no recurrence in 2 years following surgery.

## 4. Conclusion

LG-ESS presents diverse findings, including various forms of differentiation and densities, and our current case suggests that preoperative diagnostic imaging with MRI may be useful.
