# Corrigendum to “Low-Grade Endometrial Stromal Sarcoma with a Nodule-in-Nodule Appearance in Preoperative Magnetic Resonance Images”

**DOI:** 10.1155/2021/7919157

**Published:** 2021-02-08

**Authors:** Mitsuhiro Nakamura, Ryusuke Murakami, Kaoru Abiko, Taito Miyamoto, Yoshimi Kitawaki, Ken Yamaguchi, Akihito Horie, Junzo Hamanishi, Eiji Kondoh, Tsukasa Baba, Aki Kido, Sachiko Minamiguchi, Noriomi Matsumura, Masaki Mandai

**Affiliations:** ^1^Department of Gynecology and Obstetrics, Kyoto University Graduate School of Medicine, Kyoto, Japan; ^2^Department of Obstetrics and Gynecology, Japanese Red Cross Society Wakayama Medical Center, Wakayama, Japan; ^3^Department of Gynecology, Shiga General Hospital, Shiga, Japan; ^4^Department of Obstetrics and Gynecology, National Hospital Organization Kyoto Medical Center, Kyoto, Japan; ^5^Department of Obstetrics and Gynecology, Iwate Medical University Faculty of Medicine, Iwate, Japan; ^6^Department of Diagnostic Imaging and Nuclear Medicine, Kyoto University Graduate school of Medicine, Kyoto, Japan; ^7^Department of Diagnostic Pathology, Kyoto University Graduate School of Medicine, Kyoto, Japan; ^8^Department of Obstetrics and Gynecology, Kindai University Hospital, Osaka, Japan

In the article titled “Low-Grade Endometrial Stromal Sarcoma with a Nodule-in-Nodule Appearance in Preoperative Magnetic Resonance Images” [1], there was a spelling error in author Eiji Kondoh's name in the author list, where Eiji Kodoh should have read Eiji Kondoh. This is corrected as shown above.
